# Kinetic characterisation and inhibitor sensitivity of *Candida albicans* and *Candida auris* recombinant AOX expressed in a self-assembled proteoliposome system

**DOI:** 10.1038/s41598-021-94320-3

**Published:** 2021-07-20

**Authors:** Alice C. Copsey, Mario R. O. Barsottini, Benjamin May, Fei Xu, Mary S. Albury, Luke Young, Anthony L. Moore

**Affiliations:** 1 Biochemistry and Biomedicine, School of Life Sciences, University of Sussex, Falmer, Brighton, BN1 9QG UK; 2 Present Address: Theradex (Europe) Ltd, 2nd Floor, The Pinnacle, Station Way, Crawley, RH10 1JH UK; 3 Present Address: Applied Biotechnology Center, Wuhan University of Bioengineering, Wuhan, China

**Keywords:** Enzymes, Oxidoreductases, Membrane proteins, Analytical biochemistry

## Abstract

Candidemia caused by *Candida* spp. is a serious threat in hospital settings being a major cause of acquired infection and death and a possible contributor to Covid-19 mortality. Candidemia incidence has been rising worldwide following increases in fungicide-resistant pathogens highlighting the need for more effective antifungal agents with novel modes of action. The membrane-bound enzyme alternative oxidase (AOX) promotes fungicide resistance and is absent in humans making it a desirable therapeutic target. However, the lipophilic nature of the AOX substrate (ubiquinol-10) has hindered its kinetic characterisation in physiologically-relevant conditions. Here, we present the purification and expression of recombinant AOXs from *C. albicans* and *C. auris* in a self-assembled proteoliposome (PL) system. Kinetic parameters (K_m_ and V_max_) with respect to ubiquinol-10 have been determined. The PL system has also been employed in dose–response assays with novel AOX inhibitors. Such information is critical for the future development of novel treatments for Candidemia.

## Introduction

Candidemia is a major cause of morbidity and mortality in the health care system causing a serious public health risk and concomitant economic burden. The incidence of invasive fungal infections caused by *Candida* spp. has increased significantly since the 1980’s, especially amongst those who are immunocompromised and in need of critical care due to underlying illness^1^. As part of the normal microbiota of the host mucus membranes and gastrointestinal and genitourinary tracts in a healthy host the fungus is largely commensal, causing little or no damage. However, as an opportunistic pathogen when the host’s immune system is compromised these yeasts can adapt causing both superficial and life threatening infections. Consequently, they have become one of the major agents of hospital acquired infection. Whilst more than 17 different *Candida* spp. are known to cause human infection, *Candida albicans* is the most frequently isolated *Candida* spp. in a clinical setting, and is widely accepted as being the most pathogenic *Candida*^2–4^*. C. albicans* infections are the fourth most common cause of hospital acquired systemic infections in the United States with a mortality rate as high as 50%^5^.

More recently the proportion of infections caused by *C. albicans* relative to other species has decreased. The significant increase in non-*albicans* infections seen in recent years is largely the result of increasing use of prophylactic antifungal agents such as fluconazole, a drug commonly used to treat candidaemia^6^. One such species of concern is *Candida auris* which in comparison to *C. albicans* has shown reduced susceptibility to fluconazole in over 90% of cases^7^. Of particular concern is the finding that *C. auris* seems not only resistant to fluconazole, but disturbingly also resistant to the 3 main classes of antifungal drugs; azoles, echinocandins and polyenes. This species was first isolated form the ear of a Japanese patient in 2009 and is now a wide-spread pathogen which has been isolated from patients in more than 30 countries spanning all continents except Antarctica^7–9^, and alarmingly now in the UK (2016)^10^. *C. auris* infection is associated with a high mortality rate and therapeutic failure^10–15^. Of particular importance is the suggestion that patients hospitalised in ICU for Covid-19 tend to share similar risk factors and underlying co-morbities with *C. auris* infections^16^. The continuing Covid-19 pandemic may therefore provide ideal conditions for further outbreaks of *C. auris* infection in ICUs and indeed current Covid-19 patient mortality may in some way have been partly due to a contribution from *C. auris* infection^16^. Given the resistance of *C. auris* to conventional anti-fungal treatments it is therefore even more imperative that new drugs to treat this fungus are urgently developed.

Within organisms such as *C. auris* and *albicans*, the alternative oxidase (AOX) plays a very key role in facilitating continued respiratory activity and tends to be activated by stress conditions such as the presence of antifungals or oxidative inductors^17,18^. AOX is a cyanide and antimycin-resistant monotopic di-carboxylate protein which acts as terminal respiratory pathway^19,20^. AOX is ubiquitous not only amongst the plant kingdom but also found in some important plant and mammalian fungi and protist pathogens^18^. Of particular importance is the finding that AOXs are widespread amongst human parasites such as *Trypanosoma brucei* (which causes African Sleeping Sickness)^20,21^, intestinal parasites such as *Cryptosporidium parvum*^22^ and *Blastocystis hominis*^23^ and opportunistic human pathogens such as *Candida* spp.^24^. There is increasing evidence to suggest that AOX induction leads to drug tolerance in the majority of these species, and inhibition of AOX has been reported to potentiate the inhibition of the growth of *C. albicans* by fluconazole^15,24^, however to date potent and specific drugs targeted at the AOX in these organisms are yet to be achieved. Such results highlight the importance of discovering new drugs that are both well tolerated and have clearly defined biochemical targets such as the AOX.

In an attempt to investigate the extent to which *Candida* AOX proteins are sensitive to some novel AOX inhibitors we have utilised a self-assembled proteoliposome (PL) system described by Jones et. al ^25^. in which *Candida* AOX is used as the sole ubiquinol (Q_10_H_2_) oxidising agent. In our system PLs contain bacterial NADH dehydrogenase (NDH-2), ubiquinone (Q_10_), and an AOX to recycle ubiquinol to ubiquinone. NDH-2 is present in excess, such that AOX is completely rate determining and consequently the Q_10_ pool is kept fully reduced under steady-state catalysis. We demonstrate that only TAO retains enzymatic activity following solubilisation from *Escherichia coli* membranes, but activity for both *Candida* spp. is restored following incorporation into PLs confirming the importance of a lipid environment to retain structural integrity of purified AOXs. We have utilised the self-assembled Q_10_PL system to compete some novel AOX inhibitors against physiologically relevant quinol substrates thereby providing properly defined and robust inhibitor dissociation constants. We believe that such inhibitors and their analogues will prove important for the future design of anti-fungal compounds with a novel mode of action, namely targeted at the AOX which is distinct from any existing anti-fungals commercially available.

## Results

**Figure 1:**
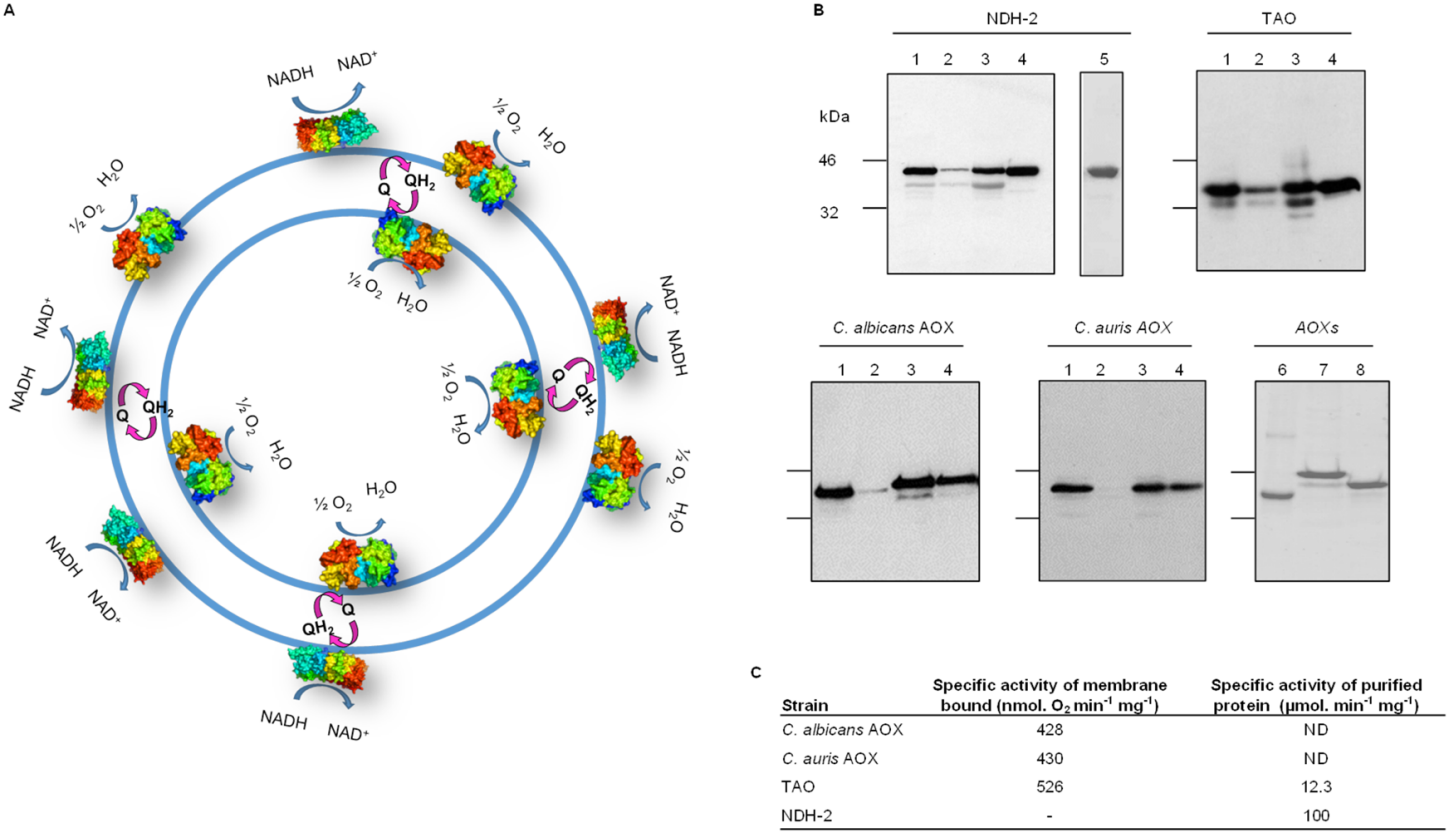
A proteolipisome system for the measurement of AOX kinetics. (**A**) PLs suitable for kinetic studies were prepared by inserting Ubiquinone-10 (Q_10_) and bacterially-expressed AOX proteins into phospholipid vesicles^26^. After PL preparation, NDH-2 was added externally to bind to the membrane of the PLs for oxidation of the electron donor NADH and subsequently Q_10_. Drawn with Microsoft Powerpoint 2016. (**B**) AOX and NDH-2 proteins were purified to homogeneity from bacterial membranes. Samples of total protein (1), insoluble (2) and soluble membrane fractions (3) and purified protein (4) were resolved by SDS-PAGE and visualised using immunoblotting (1–4) or coomassie blue staining; NDH-2 (5) TAO (6), *C. albicans* (7), *C. auris* (8). (**C**) Specific activity of membrane-bound AOX was detrmined by measurement of oxygen uptake in a Oroboros Q2k oxygraph. Specific activity of the purified AOX proteins was determined by monitoring the change of absorbance at A_278nm_ on oxidation of 100 nmol Q_1_H_2_ by the oxidase and expressed as µmol. Q_1_H_2_ min^−1^ mg^−1^. The specific activity of NDH-2 was determined by monitoring the change of absorbance at A_340nm_ on oxidation of 1 mM NADH and expressed as µmol. NADH oxidised min^−1^ mg^−1^. Note that zero activity for *C. albicans* and *C. auris* AOX was detectable (ND). Original gels and blots are shown in Supplementary Fig. 4.

To analyse the kinetic parameters of a variety of purified AOX proteins, a PL system catalysing NADH:O2 was utilised in which the ubiquinone pool was maintained reduced by an NADH dehydrogenase (NDH-2 from *Caldalkalibacillus thermarum*) and ubiquinol was reoxidised by AOX (Fig. 1A). His-tagged NDH-2 was expressed in *E. coli* C41 and purified to homogeneity as described by Heikal et al.^26^ as detailed in Materials and Methods. Figure 1B indicates that NDH-2 purified protein ran as a single band on SDS-PAGE running close to the predicted molecular mass of 44 kDa as described by Heikal et al.^25^ and typically possessed a specific activity of 100 µmol NADH oxidised min^−1^ mg protein^−1^ (Fig. 1C). Twin strep-tagged *T. brucei, C. albicans* and *C. auris* AOX were purified as outlined in Materials and Methods and visualized through both Western blotting using anti-strep antibodies and coomassie staining. Figure 1B indicates the purity of these proteins and, as predicted from their molecular weights, *C. albicans* AOX2 and *C. auris* AOX run higher than that of TAO due to the presence of a fungal specific sequence insertion^27^. All membrane-bound AOXs displayed comparable activities (Fig. 1C). However what is particularly noteworthy is the finding that even though only single bands were visualised for both *Candida* spp. no quinol oxidase activity could be detected for the purified protein with either species in contrast to the high activity observed with TAO.

### Measurement of AOX kinetic parameters

**Figure 2:**
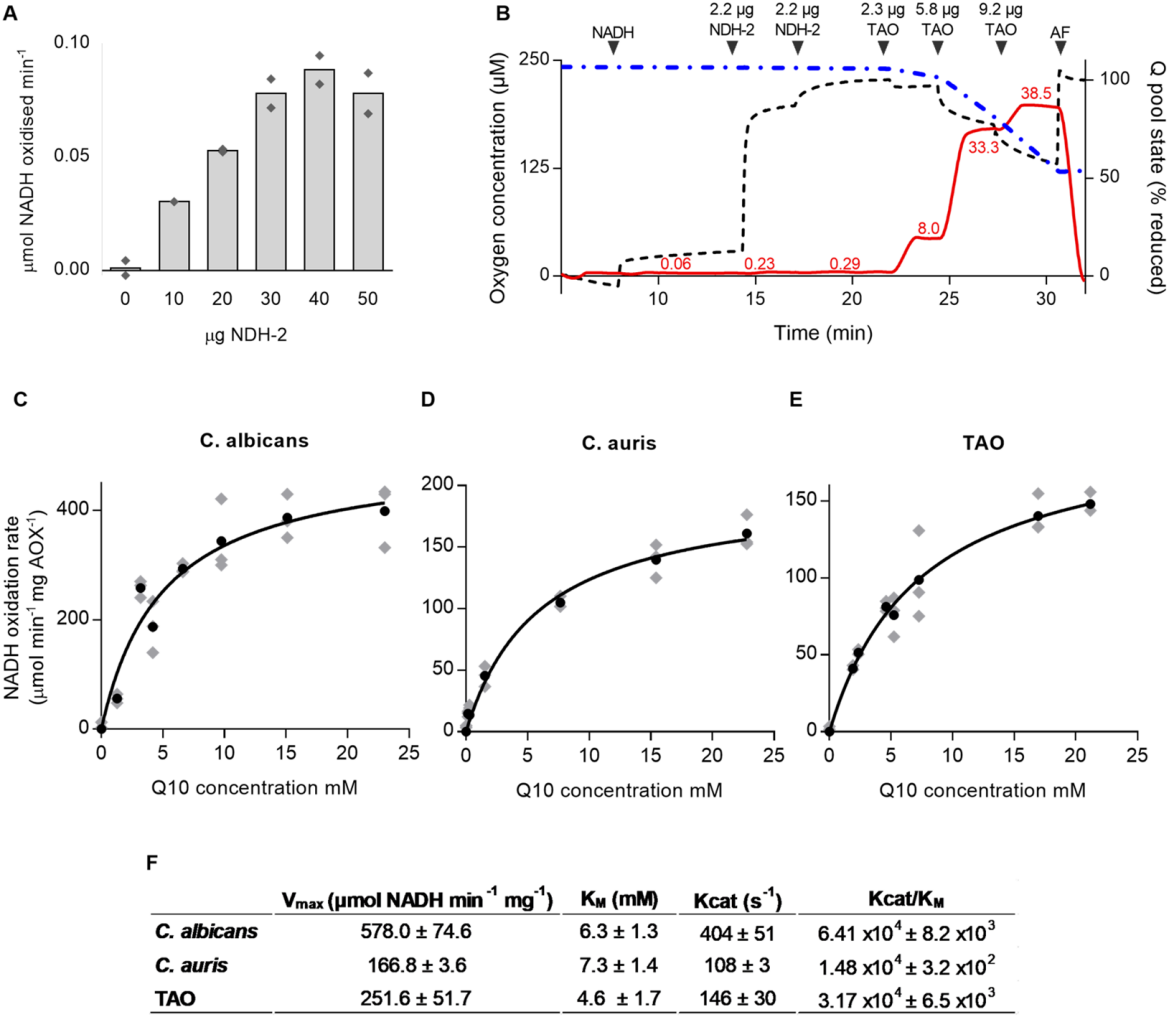
Kinetic characterization of AOX proteins incorporated into proteoliposomes with respect to ubiquinol-10 (Q_10_H_2_). (**A**) The rates of Q_10_H_2_ oxidation by bacterially-expressed AOX were calculated at increasing NDH-2 concentrations. The reaction was monitored spectrophotometrically by the oxidation of NADH by NDH-2 at A_340_ nm. Each bar shows the mean value of technical duplicates (diamonds). The point at which TAO AOX becomes rate limiting was determined and this concentration of NDH-2 protein was added to subsequent kinetic assays. (**B**) Concomitant measurements of oxygen consumption and redox poise of the Q pool in the PL system. PL prepared as described in the Methods section. The traces represent the O_2_ concentration (dot-dashed blue line) the O_2_ consumption rate (continuous red line with values in nmol O_2_ min^−1^ shown) and the redox poise of the Q pool (dashed black line). Serial additions of NADH, NDH-2, TAO and AF are indicated on top. (**C–E**) Q_10_ Michaelis–Menten curve for AOXs. The assay was performed in 200 µL 65 mM MOPS pH 7.5 with a starting concentration of 300 µM NADH. Each point (black dot) represents the mean value of individual PL preparations for which a different amount of Q_10_ was added. Technical replicates are shown as gray diamonds. The rates of reaction were plotted against the Q_10_ concentration in the membranes and fit to the Michaelis–Menten equation. Protein and Q_10_ quantifications were performed as described in the Methods section. (**F**) Summary of kinetic parameters determined for each AOX shown in panels C–E (mean ± SD).

PLs were created by incorporating approximately 50 µg of AOX protein in the presence of Q_10_ ranging from 0 to 100 nmol whereas NDH-2 protein was added directly to the activity assay at a concentration which ensured AOX remained rate limiting and this was achieved by creating the PLs in the presence of AOX and titrating AOX activity with exogenous NDH-2 until maximal rates were achieved for a pre-set ubiquinol concentration (see Fig. 2A and Supplementary Fig. 1). As AOX was incorporated during the preparation of the PLs, it is distributed on both sides of the membrane whereas since NDH-2 is added during the assay its location is restricted to the outer surface only.

We confirmed that NDH-2 maintained the Q-pool in a reduced state through the simultaneous measurement of oxygen consumption and the steady-state redox poise of the ubiquinone pool using an Oroboros NextGen-O2k high resolution respirometer fitted with a Q-electrode system^28^ the results of which are shown in Fig. 2B. It is apparent from Fig. 2B that in the absence of NDH-2 the small increase in the Q-redox poise on addition of NADH is due to a direct interaction of NADH with the glassy carbon electrode. The subsequent addition of aliquots of NDH-2, however, results in a rapid and complete reduction of the Q-pool to approximately 98% which is associated with a negligible oxygen consumption rate. Following the addition and engagement of 3 aliquots of purified TAO at this NDH-2 concentration, marked and measurable respiratory rates are observed and the Q-pool reduction level has fallen to 93%, 80 and 60% respectively. The subsequent addition of 1 µM ascofuranone (AF) completely inhibits respiration and returns the Q-pool to its fully reduced state confirming that the AOX was fully engaged under these conditions and furthermore that all oxygen consumption was due to AOX activity.

Of particular importance was the finding that incorporation of the *C. albicans* and *C. auris* AOXs into liposomes, following the rationale described for the development of this technique^25,29,30^ readily restored enzymatic activity which had been lost after solubilisation and purification. Figure 2C–E shows the Michaelis–Menten curves for AOXs from *C. albicans* (AOX2), *C. auris* and TAO respectively^25,29^. Ubiquinone concentrations in the liposome membrane were calculated assuming that 1 mg of phospholipid occupies 1 µL and hence 1 nmol of quinone in 1 mg of phospholipid corresponds to 1 mM^29,30^. In all cases typical Michaelis–Menten Kinetics were observed for all AOXs (Fig. 2C–E), the derived *K*_*m*_ and *V*_*max*_ values for each of these proteins being summarised in Fig. 2F.

It is apparent from Fig. 2F that all AOX proteins possess a similar *K*_*m*_ with respect to Q_10_ ranging from 4.6 to 7.3 mM whereas *V*_*max*_ values varied between 251–578 µmol NADH min^−1^ mg^−1^. Interestingly, the highest *V*_*max*_ (or *k*_*cat*_) was observed with *C. albicans* AOX2 at 578.0 µmol NADH min^−1^ mg^−1^ (404 s^−1^) followed by TAO at 251.6 µmol NADH min^−1^ mg^−1^ (146 s^−1^) and *C. auris* AOX at 166.8 µmol NADH min^−1^ mg^−1^ (108 s^−1^). Finally, Fig. 2F shows the catalytic efficiency or pseudo second order rate constant (*k*_*cat*_/*K*_*m*_), which reflects both substrate binding and catalytic events, for all of the AOXs incorporated into PLs. *K*_*m*_, *V*_*max*_ and *k*_*cat*_ values are statistically similar between all AOXs, however *k*_*cat*_*/K*_*m*_ differed significantly between each one (*p* < 0.001). These results suggest that *C. albicans* AOX2 has the best ability to convert substrate to product being more than double that of TAO and 4 times greater than that of *C. auris* AOX.

### AOX inhibitor titrations

The great advantage of the PL system originally described by the Hirst laboratory^25,29^ and used in this study is that AOX inhibitors can be titrated against physiologically relevant quinol substrates thereby providing properly defined and robust inhibitor dissociation constants. In this study we titrated all 3 oxidases in self-assembled PL system against Ascofuranone (AF), colletochlorins B and D (CB and CD respectively) and compared the results to that with SHAM (salicylhydroxamic acid), the most widely used AOX inhibitor. AF, CB and CD have previously been shown to behave as mixed type inhibitors against the Trypanosomal protein, with ascofuranone being effective against trypanosomes in vivo^31^ (for structures see Supplementary Fig. 2). A Q_10_ concentration of 10 mM was selected as being the closest concentration to that within the mitochondrial membrane, thereby approximating the effects of these inhibitors within a mitochondrial environment. In this case excess NDH-2 was incorporated into the PLs alongside AOX, and not added externally, in order to simplify the experimental setup and minimize handling times during dose–response assays.

Literature values for the turnover of NDH-2 and TAO are somewhat comparable, typically within the region of 15–200 s^−1^^26,32,33^ and therefore to ensure that AOX proteins were rate-limiting during IC_50_ measurements, PLs were created with a molar ratio of 400:1 NDH-2:AOX. Such a high ratio ensures that the Q-pool is maintained highly reduced and that the AOX is the rate-limiting step in the system.

**Figure 3:**
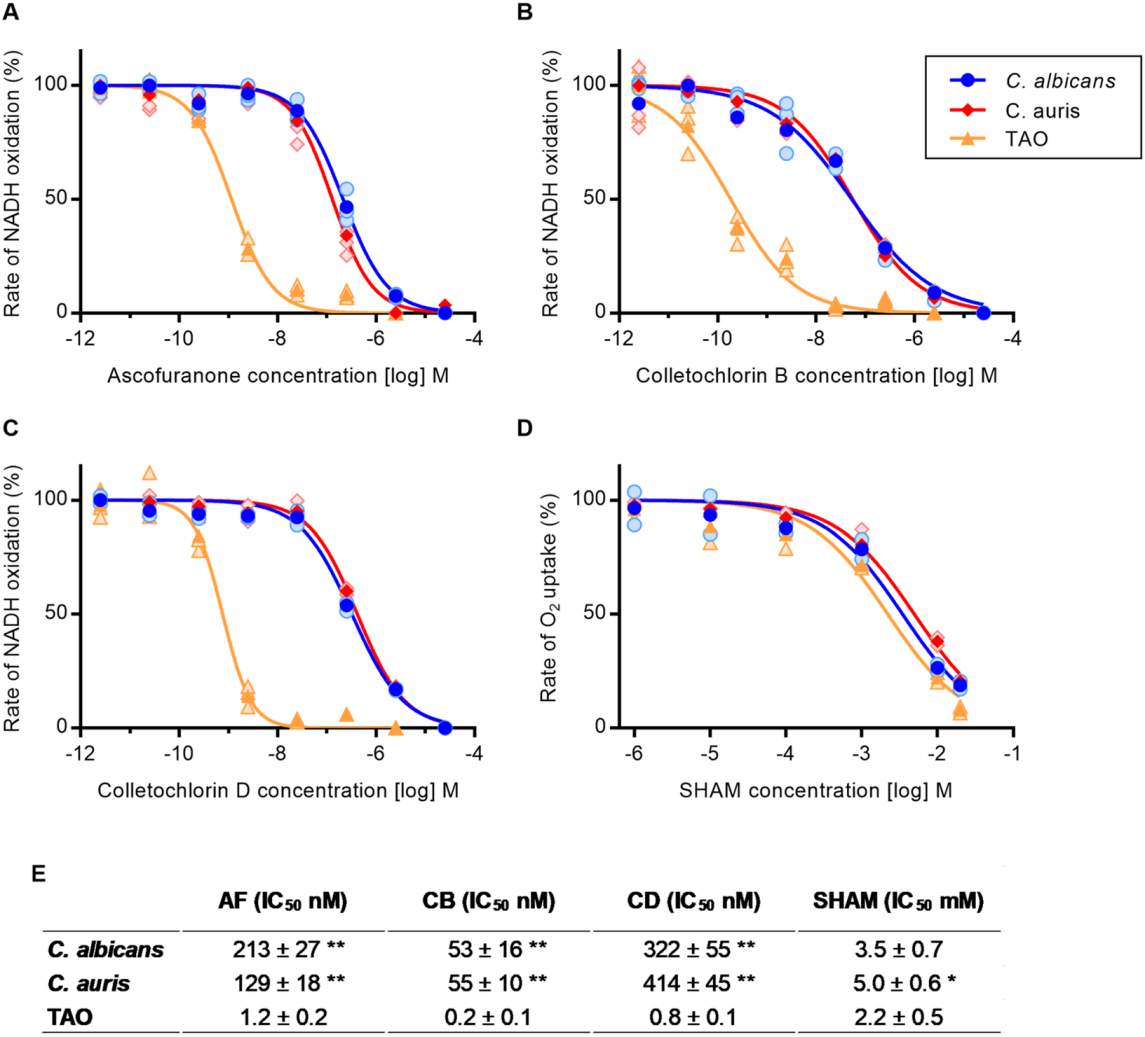
Inhibitor dose response assays for AOX incorporated into proteoliposomes*.* (**A–D**) Dose–response assay with AF, CB, CD and SHAM against AOX proteins. The assay was performed in 200 µL 65 mM MOPS pH 7.5 with a starting concentration of 300 µM NADH. Points represent the mean value (darker shading) and individual replicates (lighter shading) of technical measurements and the continuous line is the best fit for a four-parameter sigmoid function. (**E**) IC_50_ values determined from the data presented in Panels A–D. All measurements are the average of 3 biological replications ± SD. For each inhibitor, *C. albicans* and *C. auris* IC_50_ values which are significantly different from TAO (i.e., within columns) are marked with asterisks (* *p* < 0.002 and ** *p* < 0.001). *C. albicans* and *C. auris* differ significantly from each other only for AF (*p* = 0.013).

It is apparent from Fig. 3 that the three inhibitors tested were active against all of the three AOX proteins in the sub-micromolar range—much lower concentrations than SHAM which is effective only at millimolar concentrations. Indeed, SHAM is such a poor inhibitor that the high concentrations required for inhibition interfered with NADH absorbance readings and hence we resorted to oxygen consumption measurements to determine SHAM IC_50_ values. For the three AOX proteins, CB displayed the lowest IC_50_, followed by ascofuranone and CD. IC_50_ values for TAO were significantly lower than those for *C. albicans* AOX2 and *C. auris* AOX (Fig. 3E). Finally, the selectivity of ascofuranone, CB and CD towards all AOXs was evaluated in PLs containing only NDH-2, in which case DCPIP was used as an electron acceptor. Negligible effect of the inhibitors on NDH-2 up to 2.5 µM was detected (Supplementary Fig. 3).

## Discussion

AOX is a respiratory protein which is gaining increasing interest in biotechnological applications such as a possible human therapeutic agent and as a molecular target for a novel class of antimicrobial agents. It is therefore imperative to determine relevant biochemical parameters for this enzyme which will guide the future rational design of novel therapies. One such example is the development of antifungal agents effective against drug-resistant strains of *C. albicans* and *C. auris* which are major sources of hospital infections in the UK and worldwide. With the prevalence of antifungal resistance being both substantial and growing, the development of new bioactive compounds is essential, with drugs which target the switch to more virulent forms of the fungi being a prime candidate. The high resistance of *Candida* biofilms to anti-fungal compounds, in particular azoles, is one such example with the biofilms being up to 1000 times more resistant than the less virulent yeast forms^34^.

Studies aimed at preventing the switch from a yeast to the far more virulent hyphae form associated with invasive candidemia have implicated AOX as a possible therapeutic target. These include both in vitro assays using *C. albicans* which show that AOX inhibition results in oxidative stress, a reduction in cellular proliferation and an increase in suscepitbilty to fluconazole^35,36^; and multiple studies suggesting a role for AOX enzymes in activation of the cAMP-PKA signalling pathway, which is one of the signalling pathways controlling hyphae formation^37–40^. In addition a recent in vivo study using a mouse systemic model of candidiasis caused by *C. albicans* reinforced the link between AOX expression and hypha formation^41^. This study indicated that AOX enzymes are required for hypha formation upon inhibition of the classical repiratory system.

Considerable complication in the development of antifungal drugs is that the fungi are eukaryotic; this renders them harder to treat due to the obvious need to select pathogen-specific targets, and AOX is of particular interest due to its absence in mammalian electron transport chains. One challenge posed by AOX, however, is the fact that it is a monotopic membrane protein and its natural substrate is the highly hydrophobic ubiquinol-10 which is present within the phospholipid bilayer. To the best of our knowledge we present for the first time the characterization of *Candida* AOX in a PL system which is a truer reflection of the hydrophobic environment of the mitochondrial membrane rather than using detergent-solubilised AOX and the hydrophilic ubiquinol-1 analog. The advantage of the PL system is evident when one compares AOX catalytic rates before and after incorporation of pure protein into PLs. Indeed, the fungal AOXs presented here had no detectable enzymatic activity after removal of the bacterial membrane (Fig. 1C) which was promptly restored following incorporation into PLs (Fig. 2D,E). It is noteworthy that the enzymatic activity of pure AOX in solution is not a good predictor of enzymatic activity in the PL. Based on results obtained with pure (solubilised) protein, TAO has been considered to possess the highest V_max_ to date whereas fungal AOXs have shown notoriously low rates in comparison^42^. However our results demonstrate that *C. albicans* and *C. auris* AOX can also possess high V_max_ values and indeed under appropriate conditions surpass that of TAO. Moreover, AOX turnover rates obtained here are comparable to those of other mitochondrial quinol oxidases (the *cytochrome bc*_1_ complex) and a quinone reductase (complex I) in PLs, namely 620 s^−1^ and 380 s^−1^, respectively^29,43^.

Of particular importance with respect to the use of PLs is that the substrate concentration is not limited by its solubility in aqueous medium which has previously hindered the determination of the true kinetic parameters of AOX^43^. Previous attempts at characterizing AOX have used the hydrophilic ubiquinol-1 analogue which can be used only up to ca. 600 µM before forming micelles. K_m_ values for Q_1_ lie in the micromolar range of concentrations^33,42^, however our results using PLs with the natural substrate Q_10_ demonstrate that true K_m_ values for AOX are greater by 2 orders of magnitude namely 4–8 mM. The ubiquinol K_m_ value reported for the *cytochrome bc*_1_ complex (~ 10 µM) is, however, much lower than values for AOX, although it is not inconceivable that such values are possibly underestimations since the partitioning of ubiquinol into the lipid phase was not taken into consideration^43^. However, in a biological context this fits well with the AOX’s role within the mitochondria—having a significantly higher K_m_ for ubiquinol than that of the cyctochrome *bc*_1_ complex would mean that ubiquinol would preferentially bind to the latter, with the AOX only effectively being engaged as an overflow at time when the ubiquinol pool was highly reduced^21,33^.

The interest in using AOX as a therapeutic target to treat both human and plant pathogens is growing^24,35–40^, and a number of animal studies have implicated AOX for the treatment of human mitochondrial diseases using gene therapy^44–49^. The work undertaken here has shown that all 3 ascofuranone derivatives tested are promising candidates with AOX inhibition with IC_50_ values in the nM region, with colletochlorin B being the most effective. However, it should be noted that there is a significant decrease in efficacy of the compounds with the fungal AOX’s when compared to TAO, requiring a 250 fold increase in concentration. These values are reasonably consistent with those seen against other fungal AOX’s^50^, which has largely been attributed to the decrease in size of the hydrophobic cavity in the fungal species when compared to TAO being problematic for inhibitor egress^42,51^.

Novel drug-like molecules are therefore urgently required to translate the recent findings relating to AOX, its role in *C. auris* and it’s potential use as a therapeutic target for treating candidiasis particularly within a Covid-19 setting. We conclude that the incorporation of AOX into the PL system serves as an important tool not only for a more accurate characterisation of AOX catalytic rates but more importantly such a system is required to translate novel drug design currently underway in laboratories across the world into effective therapies.

## Materials and methods

### Expression and purification of NDH-2

NDH-2 protein expression and purification was based on the method of Heikal et al.^26^. Fresh *E. coli* C41 transformants carrying the plasmid pTRCndh2 were used to inoculate 10 ml starter cultures of L broth, supplemented with 100 µg ml^−1^ ampicillin, for growth over-night at 37 °C,180 rpm. Each 10 ml starter culture was used to inoculate 1 L of L-broth supplemented with 100 µg ml^−1^ carbenicillin for growth at 37 °C, 180 rpm. Once an OD_600_ of 0.5 was reached expression of NDH-2 was induced by the addition of 1 mM isopropyl-beta-D-thiogalactopyranoside (IPTG). Once growth plateaued (typically OD of 1.5 to 1.9) cells were harvested by centrifugation at 7000 × g for 15 min at 4 °C, weighed, and resuspended in lysis buffer (50 mM Tris–HCl pH 7.5, 2 mM MgCl_2_, EDTA free protease inhibitor cocktail (Roche)). The cells were lysed using two passes through a pre-cooled cell disruption system (Constant Systems Ltd) set to 30 kPa of pressure. The cell lysate was centrifuged for 15 min at 11,500 × g at 4 °C to collect cells debris and non-lysed cells.

To collect membranes, the supernatant was centrifuged for 60 min at 200,000 × g, 4 °C. Membranes were resuspended in 50 mM Tris HCl pH 8.0 at a concentration of 10 mg ml^−1^ and solubilised by drop wise addition into an equal volume of solubilisation buffer (50 mM Tris HCl pH 8.0, 2% (w/v) n-octyl-β-D-glucopyranoside (OG), 4 mM imidazole, 150 mM NaCl, EDTA free protease inhibitor cocktail (Roche)) whilst stirring at 4 °C followed by 60 min rotation at 4 °C. Finally, the sample was centrifuged for 30 min at 200,000 × g, 4 °C to collect solubilized membranes.

NDH-2-His was purified by passage through a 5 ml HiTrap TALON Crude column attached to an ÄKTA FPLC (GE Healthcare Life Sciences). The column was equilibrated at a flow rate of 1 ml per minute in binding buffer (50 mM Tris–HCl pH 8.0, 1% (w/v) OG, 2 mM imidazole, 150 mM NaCl). Protein was bound to the column at a flow rate of 0.5 ml min^−1^ and washed at a flow rate of 1 ml min^−1^ until the absorbance at A_280_ nm had returned to the baseline. Protein was eluted with elution buffer (50 mM Tris HCl pH 8, 150 mM NaCl, 400 mM imidazole and either 0.05% (w/v) n-Dodecyl β-maltoside (DDM) or 1% (w/v) OG). Peak fractions were collected in a 96 well plate. Fractions containing NDH-2 were pooled, concentrated ~ tenfold via filtration using a 30 kDa weight cut off spin concentrator, supplemented with 10% glycerol and protease inhibitors, aliquoted, flash-frozen in liquid nitrogen and stored at − 80 °C. NDH-2 activity rates were measured pre and post freezing.

### NDH-2 activity assay

The specific activity of the enzyme was measured spectrophotometrically by measuring the oxidation of NADH at A_340_ nm. The enzyme was diluted 1 in 50 in 50 mM MOPS pH 7.5 containing 250 µM Q_1_ and 300 µM NADH. Typical activity measurements were approximately 100 µmol NADH oxidized min^−1^ mg^−1^ NDH-2.

### AOX plasmid construction

The AOX from *T. brucei*, *C. albicans* and *C. auris* (GenBank accession numbers: AB070617, AF116872 and XP_018165728 respectively), lacking the mitochondrial targeting sequences (24, 26 and 24 residues respectively), were expressed in a modified version of pET15b, first described by Fedor et. al ^29^. in which the 6xHis tag was replaced with a twin-strep tag. Gene constructs were synthesised with codon optimization for expression in *E. coli* (GenScript, Piscataway, NJ), and contained *Nde*I and *Bam*HI restriction endonuclease sites at 5’ and 3’ ends respectively, for insertion into the pET15b.TS expression vector. The resulting plasmids were named pTS.TAO, pTS.CalbAOX2 and pTS.CaAOX.

### AOX-strep protein expression and membrane isolation

Over-expression of AOX was carried out in *E. coli* strain FN102^32^. There are two AOX genes in *C. albicans*, AOX1 and AOX2, whilst the former is constitutively expressed, expression of AOX2 is dependent on the growth stage and can be induced by inhibition of complex III or IV of the classical respiratory system. AOX2 was selected for these experiments.

AOX-strep was expressed in *E. coli* as described in Kido et. al.^33^. The protocol described was followed except for the following adaptions: two 50 ml day cultures of K-broth (supplemented with 100 µg ml^−1^ carbenicillin, 100 µg ml^−1^ kanamycin, 50 µg ml^−1^ 5-aminolevulinic acid (ALA), 0.2% (w/v) glucose and 50 µl per flask of metal mix (containing 0.5 g of MgSO_4_·7H_2_O, 0.25 g of FeSO_4_·7H_2_O, 0.25 g FeCl_3_ per 10 ml total volume) were inoculated with 1 ml of over-night culture (Luria broth inoculated with a single bacterial colony, supplemented with 100 µg ml^−1^ ampicillin, 100 µg ml^−1^ kanamycin, and 50 µg ml^−1^ ALA). Cultures were grown at 37 °C in a shaking incubator until OD_600_ reached approximately 0.6. Cells were collected by centrifugation, washed to remove the majority of ALA and used to inoculate 4 × 1 L of K-broth (supplemented with 100 µg ml^−1^ carbenicillin, 100 µg ml^−1^ kanamycin, 0.2% (w/v) glucose and 1 ml of metal mix) as described in the referenced protocol. The 4 L culture was incubated at 30 °C, 180 rpm, until OD_600_ reached 0.6 at which point protein expression was induced by the addition of 25 µM IPTG. Cultures were grown over-night at 30 °C, 180 rpm for approximately 13 h before harvesting cells by centrifugation at 7000 × g for 15 min, 4 °C. Cell pellets were resuspended on ice in 65 mM MOPS (KOH) pH 7.5, 1 mM MgSO_4_, 2.5 Units of benzonase plus protease inhibitors. The cells were lysed using two passes through a pre-cooled cell disruption system (Constant Systems Ltd) set to 30 kPa of pressure. The cell lysate was centrifuged for 15 min at 11,500 × g at 4 °C to collect cells debris and non-lysed cells followed by 60 min, 200, 000 × g at 4 °C to collect the membrane fraction.

### AOX-strep protein solubilisation and purification

Isolated membranes were resuspended in 65 mM Mops pH 7.5 at a concentration of approximately 10 mg ml^−1^ and solubilised by drop wise addition into an equal volume of solubilisation buffer (65 mM MOPS (KOH) pH 7.5, 2% (w/v) fos-choline 12 (FC12), 400 mM MgCl_2_, 40% glycerol, with protease inhibitors) whilst stirring at 4 °C followed by 60 min rotation at 4 °C. The sample was centrifuged for 30 min at 200,000 × g, 4 °C to collect solubilized membranes.

AOX-strep was purified by passage through a 5 ml StrepTrap HP column attached to an ÄKTA FPLC. The column was equilibrated at a flow rate of 1 ml min^−1^ in binding buffer (65 mM MOPS (KOH) pH 7.5, 1% (w/v) FC12, 50 mM MgSO_4_, 160 mM NaCl, 20% glycerol). Protein was bound to the column at a flow rate of 0.5 ml min^−1^ and washed at a flow rate of 1 ml min^−1^ until the A_280_ nm had returned to the baseline. Protein was eluted with elution buffer (65 mM MOPS (KOH) pH 7.5, 2.5 mM desthiobiotin, 0.042% DDM, 50 mM MgSO_4_,160 mM NaCl, 20% glycerol), and peak fractions were collected in a 96 well plate. Fractions containing AOX were pooled, concentrated ~ tenfold via filtration using a 30 kDa weigh cut off spin concentrator, aliquoted, flash-frozen in liquid nitrogen and stored at − 80 °C.

### AOX activity assay

The specific activity of the purified protein was measured spectrophotometrically by measuring the oxidation of Q_1_H_2_ at A_278_ nm. The activity was measured in 50 mM MOPS (KOH) pH 7.5 containing 150 µM Q_1_H_2_.

### Western blotting

Proteins were separated by SDS-PAGE, transferred to nitrocellulose membranes, and visualised with the appropriate antibodies followed by HRP-conjugated secondary antibodies (DAKO, 1:2000). HRP activity was detected using Pierce ECL Western Blotting Substrate followed by exposure to Amersham Hyperfilm. Antibody dilutions used for Western blotting were: anti-AOX (AOA)^52^ 1:1000 and anti-StrepII (IBA: 2–1509-001) 1: 20,000. The anti-Strep antibody is conjugated directly to HRP.

### Preparation of proteoliposomes

PLs were prepared exactly as described in previously published protocols using phospholipids; phosphatidylcholine, phosphatidylethanolamine and cardiolipin, mixed at a ratio of 8:1:1^25,29^. A typical preparation used a total of 10 mg phospholipids and 100 nmol of Q_10_, both of which are essential for PL activity^50^. The only modifications to the protocol were the detergent used to partially solubilise the phospholipid/Q_10_ mixture (0.5% DDM in our preparations) and the protein content of the phospholipid vesicles as follows. For IC_50_ measurements, 5 µg of AOX protein and 500 µg of NDH-2 were incorporated into the phospholipid vesicles. 100 nmol of Q_10_ were added whilst preparing the liposome vesicles for IC_50_ measurements.

For measurement of K_m_ and V_max_ 50 µg of AOX protein was incorporated into the phospholipid vesicles. NDH-2 protein was omitted from the PL preparation and instead added directly to the activity assay at a concentration to ensure the AOX was rate limiting. A typical preparation resulted in a ratio of 1:100:200 AOX:phospholipid:NDH-2. Between 0 and 160 nmol of Q_10_ were added whilst preparing the liposome vesicle for the measurement of K_m_ and V_max_.

### Quantification of protein, Q_10_ and lipid incorporation into the phospholipids

Protein and Q_10_ quantification were performed as described in previously published protocols^29^. The quantification of both protein (using the Amido black assay) and Q_10_ (by HPLC) was performed after the completion of the experiments to ensure any loss of protein or Q_10_ during PL preparation did not effect these measurements. Lipid content was determined using the AMES test as described in previously published protocols^25^.

### Activity measurements in proteoliposomes

Activity assays were carried out in solutions containing 65 mM MOPS pH 7.5 and 300 µM NADH. AOX was inhibited, when required, by addition of AOX inhibitors at final concentrations ranging from 2.5 pM to 25 µM for all inhibitors except for SHAM where the highest concentration was 500 µM. The selectivity of those inhibitors was evaluated with PLs containing only NDH-2 for which 300 µM 2,6-dichlorophenolindophenol (DCPIP) was used in the reaction medium as an electron acceptor instead of AOX. Rates of NADH oxidation were measured spectrophotometrically at A_340_ nm using a Multiscan Sky 96-well plate reader (Thermo Scientific) and baseline rates from the reaction components alone (i.e. in the absence of any enzyme) were subtracted. Additionally, SHAM dose–response assays were performed in the high resolution oxygraph Oroboros Q2k (NextGenO2k, Oroboros Inc.) at concentrations as high as 20 mM by monitoring the PL oxygen consumption rate. The Oroboros was selected for SHAM dose–response assays due to the absorbance spectrum of the compound overlapping with the NADH signal. All other assays were performed using the plate reader for high throughput and to reduce reagent use.

### Ubiquinone redox poise measurement

Simultaneous measurement of oxygen consumption and the ubiquinol/ubiquinone ratio was performed in a Oroboros O2k with a glassy carbon and platinum electrodes^28^ in 65 mM MOPS (KOH) pH 7.5 and 2 µM Q_2_. 5 µL of a PL prepared without protein (only lipids and 100 nmol Q_10_) were added in to the 2 ml measurement chamber followed by NADH, NDH-2, AOX and ascofuranone at concentrations described within the results section.

### Statistics and reproducibility

Measurements were performed in technical triplicate and each experiment was performed three independent times (e.g. distinct protein purification batches) unless otherwise stated and similar results were obtained each time. No data points were excluded from the analyses. Data fitting and plotting were performed with GraphPad Prism 7.0 (GraphPad Software Inc.). AOX Kinetic parameters and IC_50_ values were evaluated with the 2-way ANOVA test followed by Tukey’s multiple comparison test.

## Supplementary Information

Supplementary Information 1.

Supplementary Information 2.

## Data Availability

All data used in this study are included within the main text or provided in the supplementary files. Source data for graphs/charts in the main figures is available in Supplementary Data 1. Any additional information and biological material described in the study is available from the corresponding authors upon request.
