# Supplementary material for: Kinetic characterisation and inhibitor sensitivity of Candida albicans and Candida auris recombinant AOX expressed in a self-assembled proteoliposome system
Source: Sci Rep. 2021 Jul 20;11:14748. doi: 10.1038/s41598-021-94320-3 (PMC8292455; doi:10.1038/s41598-021-94320-3)
Supplement: Supplementary file 1 — Supplementary Information 1. [file 41598_2021_94320_MOESM1_ESM.docx]

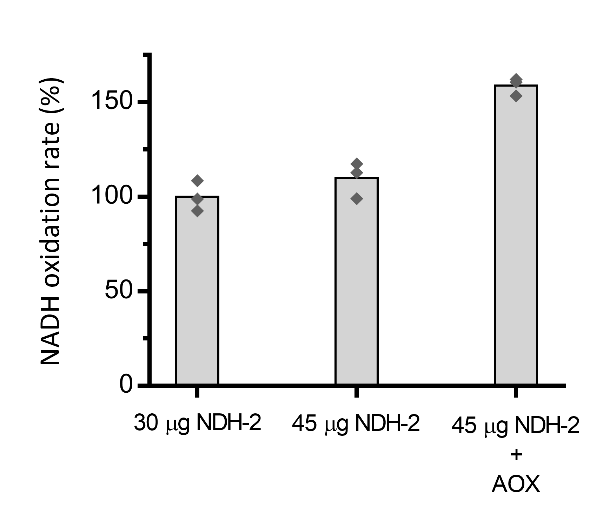


**Supplementary Figure 1.** NADH oxidation rate of PLs prepared with TAO as described in the Materials and Methods after external addition of 30 µg pure NDH-2, 45 µg pure NDH-2 or 45 µg NDH-2 plus 2µg AOX. It is demonstrated that AOX is the rate-limiting enzyme in this system.

**Supplementary Figure 1**

**Supplementary Figure 2. Structures of the AOX inhibitors.**


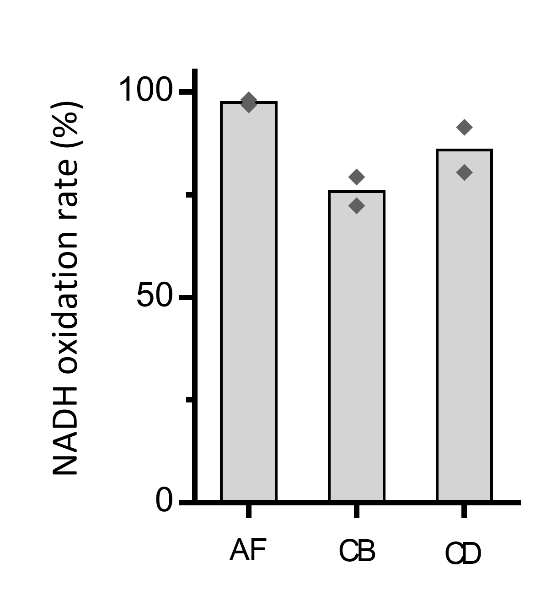


**Supplementary Figure 3.** **Effect of AOX inhibitors on NDH-2 activity.** Testing of AOX inhibitors on NDH-2. PL were prepared with NDH-2 as described in Materials and Methods and assayed using DCPIP as electron acceptor, and assayed in the presence of 2.5 µM ascofuranone (AF), colletochlorin B (CB) or colletochlorin D (CD). Non-inhibited PL was used to determine the inhibited (i.e. 100% activity) rate.


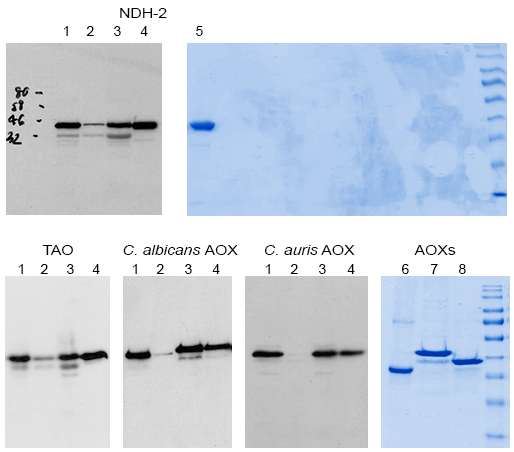


**Supplementary Figure 4.** **Uncropped blots and gels of NDH-2 and AOX.** Lane identification is exactly the same as in the main text.
